# The rhodopsin-retinochrome system for retinal re-isomerization predates the origin of cephalopod eyes

**DOI:** 10.1186/s12862-021-01939-x

**Published:** 2021-11-29

**Authors:** Oliver Vöcking, Lucas Leclère, Harald Hausen

**Affiliations:** 1 Sars International Centre for Marine Molecular Biology, University of Bergen, Thormøhlensgate 55, 5008 Bergen, Norway; 2 Sorbonne Université, CNRS, Laboratoire de Biologie du Développement de Villefranche-sur-Mer (LBDV), Villefranche-sur-Mer, France; 3 Present Address: Department of Biology, University of Kentucky, Thomas Hunt Morgan Building, 675 Rose Street, Lexington, KY 40508 USA

**Keywords:** Opsin, Retinochrome, RALBP, RLBP1, Visual cycle, Mollusk, Photoreceptor

## Abstract

**Background:**

The process of photoreception in most animals depends on the light induced isomerization of the chromophore retinal, bound to rhodopsin. To re-use retinal, the all-trans-retinal form needs to be re-isomerized to 11-cis-retinal, which can be achieved in different ways. In vertebrates, this mostly includes a stepwise enzymatic process called the visual cycle. The best studied re-isomerization system in protostomes is the rhodopsin-retinochrome system of cephalopods, which consists of rhodopsin, the photoisomerase retinochrome and the protein RALBP functioning as shuttle for retinal. In this study we investigate the expression of the rhodopsin-retinochrome system and functional components of the vertebrate visual cycle in a polyplacophoran mollusk, *Leptochiton asellus,* and examine the phylogenetic distribution of the individual components in other protostome animals.

**Results:**

Tree-based orthology assignments revealed that orthologs of the cephalopod retinochrome and RALBP are present in mollusks outside of cephalopods. By mining our dataset for vertebrate visual cycle components, we also found orthologs of the retinoid binding protein RLBP1, in polyplacophoran mollusks, cephalopods and a phoronid. In situ hybridization and antibody staining revealed that *L. asellus* retinochrome is co-expressed in the larval chiton photoreceptor cells (PRCs) with the visual rhodopsin, RALBP and RLBP1. In addition, multiple retinal dehydrogenases are expressed in the PRCs, which might also contribute to the rhodopsin-retinochrome system.

**Conclusions:**

We conclude that the rhodopsin-retinochrome system is a common feature of mollusk PRCs and predates the origin of cephalopod eyes. Our results show that this system has to be extended by adding further components, which surprisingly, are shared with vertebrates.

**Supplementary Information:**

The online version contains supplementary material available at 10.1186/s12862-021-01939-x.

## Background

Rhodopsin is fundamental for light detection in animals. In most cases, it consists of a protein part, opsin and a chromophore, 11-cis-retinal [1, 2]. The basic mechanism of light detection is the same for all known kinds of rhodopsins. Upon photon absorption, 11-cis-retinal isomerizes to all-trans-retinal leading to a conformational change of the opsin, which in turn activates a G-protein. After a short period, the opsin is deactivated and has to return to the initial state bound again to 11-cis retinal, before it can be activated another time. Several pathways have been described fulfilling this task and differ considerably between organisms and kind of opsin [3].

The visual cycle in rods and cones of the vertebrate retina, is the most complex and best studied visual system in animals. Upon deactivation, the light sensing rhodopsin, a member of the c-opsin type of visual pigments, releases the all-trans retinal and is in turn supplied with newly synthesized or re-isomerized 11-cis retinal [4]. Re-isomerization of the retinal requires multiple steps. The retinal is first actively transported into cells of the retinal pigment epithelium (RPE) or Müller cells and then stepwise modified involving chaperoning retinal binding proteins acting as transport modules, retinal dehydrogenases (RDHs) and certain isomerases before the final product 11-cis retinal is transported back into the PRCs [see [4]]. No light is required for the pathway (thus also called dark cycle), but light may nevertheless influence the process by action of RGR, a specialized opsin type expressed in RPE and Müller cells. RGRs are not able to induce phototransduction but are known to preferentially bind retinal in the all-trans conformation and to have in vitro the ability to photoisomerize all-trans-retinal directly to 11-cis-retinal [5, 6].

Biochemical and phylogenetic analyses suggest that the vertebrate dark cycle of retinal re-isomerization is a vertebrate innovation [7–9]. Indeed, the two key enzymes responsible for all-trans-retinol to 11-cis-retinol re-isomerization—Retinol acyltransferase (Lrat) and Isomerohydrolase (RPE65)—were shown to be restricted to vertebrates [9]. Other visual cycle elements were nevertheless found outside vertebrates, notably Retinal dehydrogenases (RDHs) [10, 11] and Retinaldehyde-binding protein (RLPB1); the latter was however only found in *Hydra* outside of chordates [7].

Opsin reinitialization in the eyes of protostomes seems to be very different and much simpler than in vertebrates. In cephalopods and arthropods, retinal remains bound to the r-opsin and is re-isomerized by light-dependent photoconversion, simply by absorption of another light quant [4, 12, 13]. Accordingly, rhodopsins with such capability are classified bistable, while those lacking this feature, like c-opsins in vertebrate rods and cones, are called monostable [14, 15].

Evidence is increasing that other steps complement the photoconversion in PRCs with bistable pigments. In insects, enzymatic processing by retinal dehydrogenases also plays an important role in retinal recycling. If vitamin A, the base needed to produce new retinal, was cut out of the diet of *Drosophila*, knocking down *PDH* (a member of the RDH family) resulted in a decrease of retinal availability in PRCs [10, 11].

In squid eye PRCs the bistable r-opsin is accompanied by another bistable opsin, retinochrome, which can catalyze a transition from all-trans-retinal to 11-cis-retinal by absorbing light but does not induce phototransduction [16]. The squid retinochrome is considered phylogenetically related to vertebrate peropsins and RGRs [2]. Pioneering studies on the function of cephalopod retinochrome uncovered the functional involvement of a retinal binding protein RALBP in cephalopod retinal recycling [16, 17]. RALBP is able to bind retinal in both conformations and may thereby serve as a shuttle transporting all-trans-retinal from r-opsin to retinochrome and 11-cis-retinal the other way round [16, 18]. The advantage of employing a second bistable opsin in this manner may be multifaceted: more efficient retinal re-isomerization and forming a storage capacity of retinal in dark conditions [4, 19].

The rhodopsin-retinochrome system was for a long time only known from cephalopods. First evidence for a broader taxonomic distribution came by the proposed co-expression of retinochrome and RALBP in the stalk eye of gastropods based on immunostainings using anti-squid antibodies [19–21]. However, phylogenetic analyses of the respective gastropod protein sequences are still missing and only recently genomic and RNA-seq data hinted for a broader distribution of squid retinochrome orthologs [22].

We investigate the expression and phylogenetic origin of visual cycle elements in the larval eyes of the chiton *Leptochiton asellus*, a mollusk, which is distantly related to cephalopods. While the phylogeny of mollusks is not yet fully resolved, recent phylogenetic studies suggest that the last common ancestor of chitons and cephalopods is the last common ancestor of most if not all living mollusks [23–26].

*L. asellus* develops eyespots during their larval life in an unusual position (posterior to the prototroch in the trochophore larvae) but despite this peculiarity, the molecular identity of these eyes resembles that of other marine invertebrate larvae [27–31]. Adult stages in some chiton species possess eye-like structures in their anterior shells, called aesthetes, equipped with lenses and photoreceptor cells that have been a matter of research interest [32–34]. However, it was assumed that the larval nervous system including the larval eyes do not remain after metamorphosis and play no further role in adult life [35]. Contrary to this, studies suggested there might still be remnants of the larval eyes in the adult of some species [27, 36, 37]. Morphological studies in different species have shown that photoreceptor cells within the larval eyes are of mixed morphology, combining cilia and microvilli in the same cell [29, 30, 38]. These PRCs express two kinds of visual opsins, an r-opsin and a member of the newly described xenopsins [30]. This raises the question, whether more than one mechanism of retinal re-isomerization may be implemented in the PRCs of these animals.

This study aims to decipher whether the squid rhodopsin-retinochrome system is also active in the eyes of non-cephalopod mollusks and whether elements involved in retinal re-isomerization in vertebrates and arthropods are also present in lophotrochozoans.

We found strong evidence that the rhodopsin-retinochrome system complementing photoreisomerization of bistable opsins is not only present in cephalopods, but rather a solution generally applied in mollusks and possibly also other protostomes. Our data corroborate that vertebrate RGRs and protostome retinochromes may both be involved in visual cycling and we propose an independent loss of the capability to evoke phototransduction in these two opsin families. Further, we identified for the first time in protostomes retinal binding protein-1 (RLBP1), a component of the vertebrate dark cycle and show that the chiton ortholog is specifically expressed in photoreceptor cells. We show that retinal dehydrogenases are also expressed in the chiton eye, but whether they are orthologs to insect and vertebrate eye retinal dehydrogenases remains elusive. Taken together, our data suggest that retinal re-isomerization in mollusks follows a common theme, is more complicated than hitherto anticipated, and possibly involves elements of the vertebrate dark cycle. Hence, the findings may provide first hints on a common origin of certain steps in rhodopsin reinitialization in animal eyes.

## Results

### Retinochrome is present in various protostomes

**Fig. 1:**
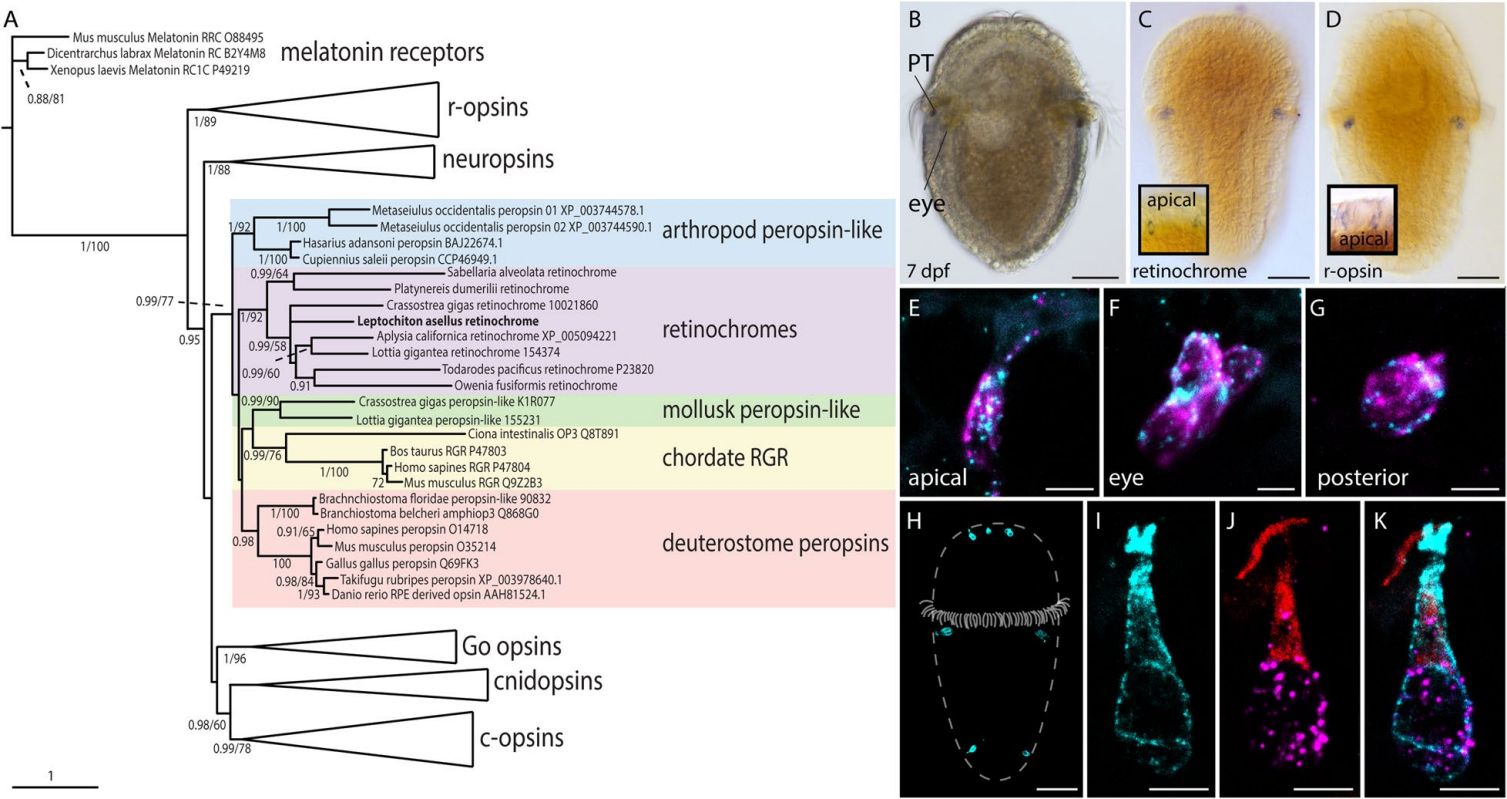
Expression and protein distribution of retinochrome in 7 dpf *Leptochiton asellus* larvae and evolution of the photoisomerase opsin family. **A** Maximum-likelihood tree (RAXML) of the photoisomerase opsin family. The photoisomerases form a well-supported group and therein the subgroups arthropod peropsin-like pigments, chordate RGRs, deuterostomes peropsins, retinochromes containing Las-retinochrome, and mollusk peropsin-like pigments. Bayesian posterior probabilities (left value) higher than 0.90 and ML bootstrap (500 replicates, right value) higher than 50% are indicated below or above branches (see SI for uncollapsed tree). **B** 7 dpf larva with eyespot under the prototroch (PT). **C** Expression of *Las-retinochrome* in the eye and apical PRCs (inlet). **D** *Las-r-opsin* expression in the eye and the apical PRCs (inlet). **E**–**G** *Las-retinochrome* (cyan) and *Las-r-opsin* (magenta) are co-expressed in all PRC of *L. asellus* in the apical area (**E**), the eye PRCs (**F**) and the posterior PRCs (**G**). **H** A specifically designed antibody against Las-retinochrome is expressed in all PRCs of 7 dpf larvae, in the apical area, the eyes and the very posterior end. **I–K** The combination of *Las-r-opsin *in situ hybridization and stainings with specifically designed antibodies against Las-r-retinochrome and Las-r-opsin reveals the cellular distribution of the opsin proteins. Las-retinochrome protein (cyan) is distributed throughout the cell body and the apical part of the cell (**I**), whereas Las-r-opsin protein (red) is restricted to the apical surface and *Las-r-opsin* mRNA (magenta) to the nucleus (**J**). Las-retinochrome protein reaches the cell surface but does not expand apically like Las-r-opsin protein (**K**). Scale bars 100 μm in **B**–**D**,**H**; 2,5 μm in **E**–**G**; 5 μm in **I**–**K**

Mining our *L.asellus* RNA-seq data, we identified a sequence bearing high resemblance to the squid retinochrome. We extended the search and screened our own transcriptome and publicly available data in order to analyze the evolution of vertebrate and invertebrate peropsins, RGRs, and retinochromes, which are often referred to as photoisomerase family [39, 40]. We chose representatives of other opsin families and melatonin receptors as outgroup and performed both, maximum likelihood (ML) and Bayesian phylogenetic analyses (Fig. 1A, Additional file 1: Fig. S1). As a result, monophyly of chordate RGRs, deuterostome peropsins and arthropod peropsin-like opsins is well supported (posterior probabilities PP ≥ 0.98). A number of protostome sequences, including *Las-retinochrome* and the squid retinochrome, form a well-supported retinochrome group (PP = 1) (Fig. 1, Additional file 1: Fig. S1) corroborating recently published opsin analyses by Ramirez et al. [22] and Vöcking et al. [30]. Two sequences of the mollusks *Lottia gigantea* and *Crassostrea gigas* form a separate group in our analysis and are referred to as mollusk peropsin-like opsins by Ramirez et al. [22]. The interrelation of the mentioned groups, however, remains uncertain.

**Fig. 2:**
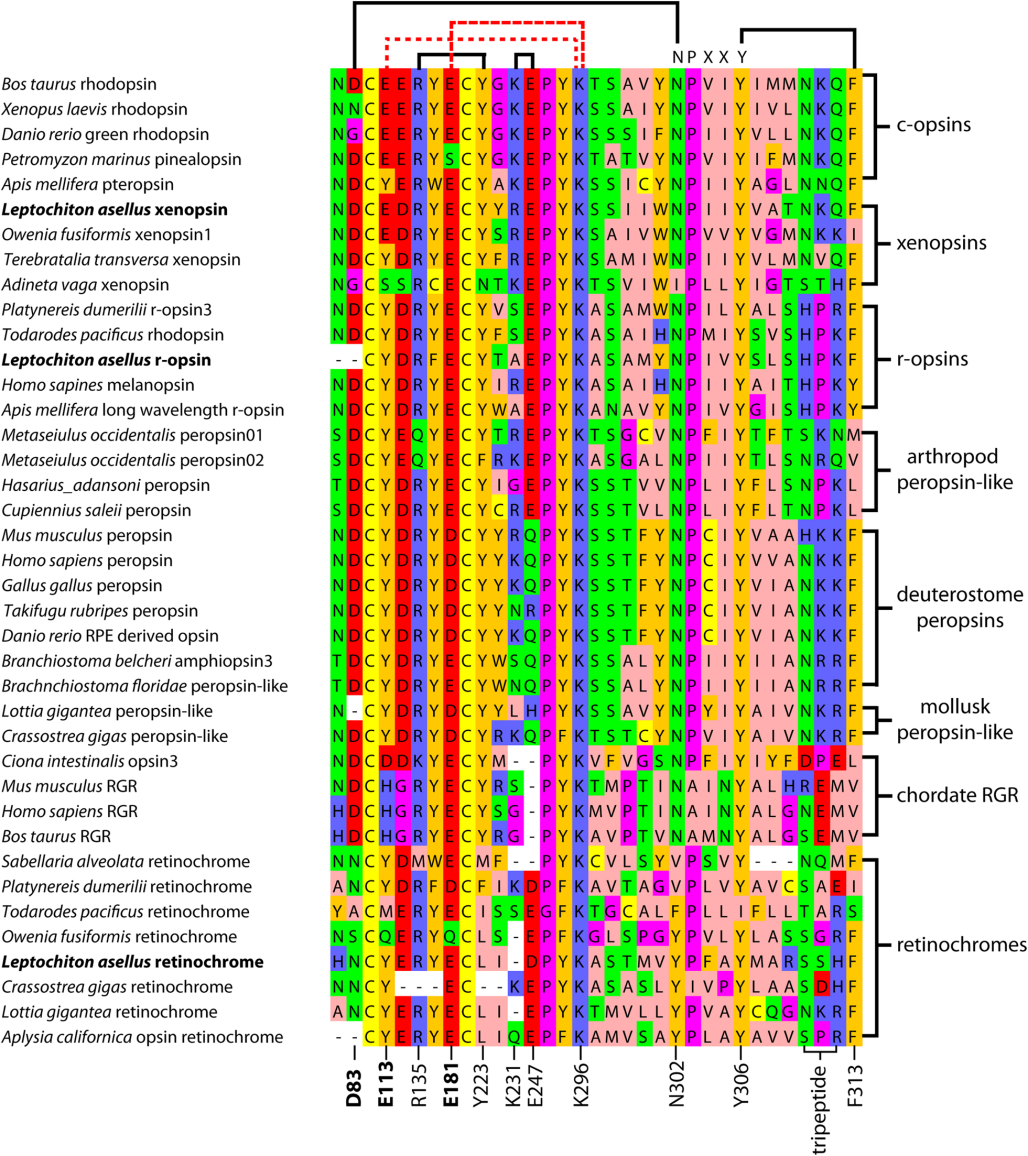
Different motifs enrolled in stabilizing the active rhodopsin state and G-protein activation. Pairwise interaction of D83/N302, R135/Y223, K231/E247 and Y306/F313 as well as NPXXY motif are highly conserved in signaling rhodopsins. Compared to c-opsins and r-opsins, deuterostome peropsins are most conserved, while chordate RGRs and retinochromes show most deviations. E113 acts as a counterion in monostable visual pigments and is well conserved among canonical c-opsins. Only in xenopsins it is conserved in a significant scope, as in the sequences of *L. asellus* and *O. fusiformis*

The placement of *L. asellus* retinochrome in a clade with squid retinochrome suggests similar biochemical properties. Hence, we compared the positions of amino acids with known importance for protein function. Particularly, we focused on candidates that differ in mono- and bistable opsins. While the negatively charged amino acid E113 is known to act as counter-ion stabilizing retinal binding in monostable opsins, this position is occupied by an uncharged amino acid in bistable opsins [15, 39]. In consequence, sequence analyses have been used to predict mono- or bistability of opsins, though reliability has been questioned [39, 41]. Analysis of the Las-retinochrome sequence shows that this opsin exhibits an uncharged residue at position 113 as do squid retinochrome and many other opsins with proven or presumed bistability as well as all photoisomerase family sequences included in this study (Fig. 2). Las-retinochrome may thus have the potential to re-isomerize all-trans retinal as do squid retinochromes [42].

### Expression and cellular distribution of *Las-retinochrome* in larval PRCs

A potential role of Las-retinochrome in re-isomerization of retinal for light detection, requires expression in (as in the squid) or close (as RGRs in vertebrates) to PRCs. Using in situ hybridization, we found *Las-retinochrome* to be localized in the eye region, as well as in some cells in the apical area and two cells at the very posterior end of the larvae (Fig. 1B, C). In fact, this expression pattern is highly similar to the expression of *r-opsin* (Fig. 1D) found in *L. asellus* larvae [31]. In order to check for cellular co-expression, we performed double in situ hybridization experiments combining *Las-retinochrome* and *Las-r-opsin*. The data show overlapping expression of both opsins in all PRCs, in the apical area (Fig. 1E), the eyes (Fig. 1F) and the very posterior end of the larvae (Fig. 1G). To also localize the opsin protein, we designed custom-made specific antibodies against Las-retinochrome*.* The respective antibody staining and the *Las-retinochrome *in situ hybridization show the same pattern (Fig. 1 H), mutually confirming the results. Combining custom-made antibodies against Las-r-opsin and Las-retinochrome with *Las-retinochrome *in situ hybridization, finally revealed subcellular localization of the transcript and the opsin proteins. As expected, the *Las-retinochrome* transcript is localized in the cell body around the nucleus (Fig. 1I). In contrast, Las-r-opsin protein is found in the apical area of the PRC (Fig. 1J, K) only corroborating earlier results [31], whereas Las-retinochrome is distributed throughout the apical part and the cell body as well (Fig. 1I, K), similarly to previously described retinochrome and r-opsin distribution in cephalopod eye PRCs [43]. The Las-r-opsin protein expands on the cell surface matching the course of the sensory microvilli of these cells [31], whereas the Las-retinochrome signal reaches the cell surface but does not expand like the Las-r-opsin signal (Fig. 1I–K).

### Different motifs enrolled in G-protein activation are modified in protostome retinochromes and vertebrate RGRs

The key function of opsins is to initiate phototransduction after light detection by activating a G-protein. However, members of the opsin photoisomerases differ in this context because at least partly they appear to have lost the ability to interact with G-proteins. Numerous functional studies were undertaken (mainly on *Bos taurus* rhodopsin) and revealed that certain amino acids are critical for the interaction of the activated rhodopsin and G-protein. This includes pairwise interaction of D83/N302, R135/Y223, K231/E247 and Y306/F313 as well as the highly conserved NPXXY motif in G-protein coupled receptors [44–48]. We analyzed conservation of these residues in the photoisomerase family included in this study (Fig. 2). Compared to signaling rhodopsins such as c-opsins and r-opsins, deuterostome peropsins are most conserved and show deviations only in K231/E247. The same holds true for the mollusk-peropsin-like group, though one sequence also shows deviations in D83/N3012. In difference, in arthropod peropsin-like pigments deviations occur in Y306/F313 and partly R135/Y223. Strongest deviations occur in chordate RGRs and retinochromes. These are the only groups deviating from the highly conserved NPXYY motif. Notably, there is no common pattern. While N302 is modified in retinochromes, P303 is modified in chordate RGRs. In addition, chordate RGRs show severe modification of K231/E247, that latter amino acid completely missing, and in Y306/F313, while retinochromes show deviations R135/Y223 and D83/N302.

### A third rhodopsin expressed in *Leptochiton asellus* PRCs may be monostable

We previously showed that *L. asellus* PRCs employ another opsin, which belongs to a newly described group called xenopsins [22, 30]. The shown co-expression with Las-r-opsin makes Las-xenopsin another potential target of visual cycling in *L. asellus* PRCs. Against this background, we studied the sequence residues of xenopsins characterizing mono- and bistable rhodopsins, respectively. Notably, E113 acting as counter-ion in the monostable rhodopsins, occurs outside vertebrate rhodopsins only in several xenopsin sequences. Since Las-xenopsins exhibits this feature, it might indeed be monostable. In difference to Las-retinochrome, presence of the critical residues for G-Protein activation indicate a signaling function (Fig. 2).

### Two retinal binding proteins identified in *Leptochiton asellus*

Mining our *L. asellus* RNA-seq transcriptome data revealed a sequence highly similar to the squid RALBP. Surprisingly, we also identified an additional sequence. A blast search (blastp) against this sequence revealed similarities to a separate group of retinal binding proteins so far only known from chordates, named RLBP1 (= cellular retinal binding protein CRALBP) [49].

In order to analyze the affiliation of the *L. asellus* sequences, we performed an extensive search on sequences exhibiting the CRAL_TRIO domain with special focus on sequences similar to vertebrate RLBP1 and cephalopod RALBP across metazoan publicly available data complemented by our own RNA-seq data. Since proteins with this domain are known to fulfill various functions, numerous candidates were found in all major animal groups. Five transcripts containing the CRAL_TRIO domain were found in the *L. asellus* data set. Phylogenetic analyses support the existence of many different evolutionary old groups containing CRAL_TRIO domains, including members from diverse taxa (Additional file 2: Fig. S2). Cephalopod RALBP fell into a well-supported group containing *Las-RALBP*, sequences from other mollusks and several annelid sequences. The resolution of the tree did not allow drawing conclusions concerning the precise evolutionary relationships between RALBP and homologs in deuterostomes. Interestingly, these analyses also grouped one *L. asellus* sequence (*Las-RLBP1*) and two *Octopus bimaculoides* sequences with known chordate and *Hydra* RLBP1 with high support. These analyses provide, to our knowledge, the first evidence of RALBP outside cephalopods and RLBP1 in protostomes.

**Fig. 3:**
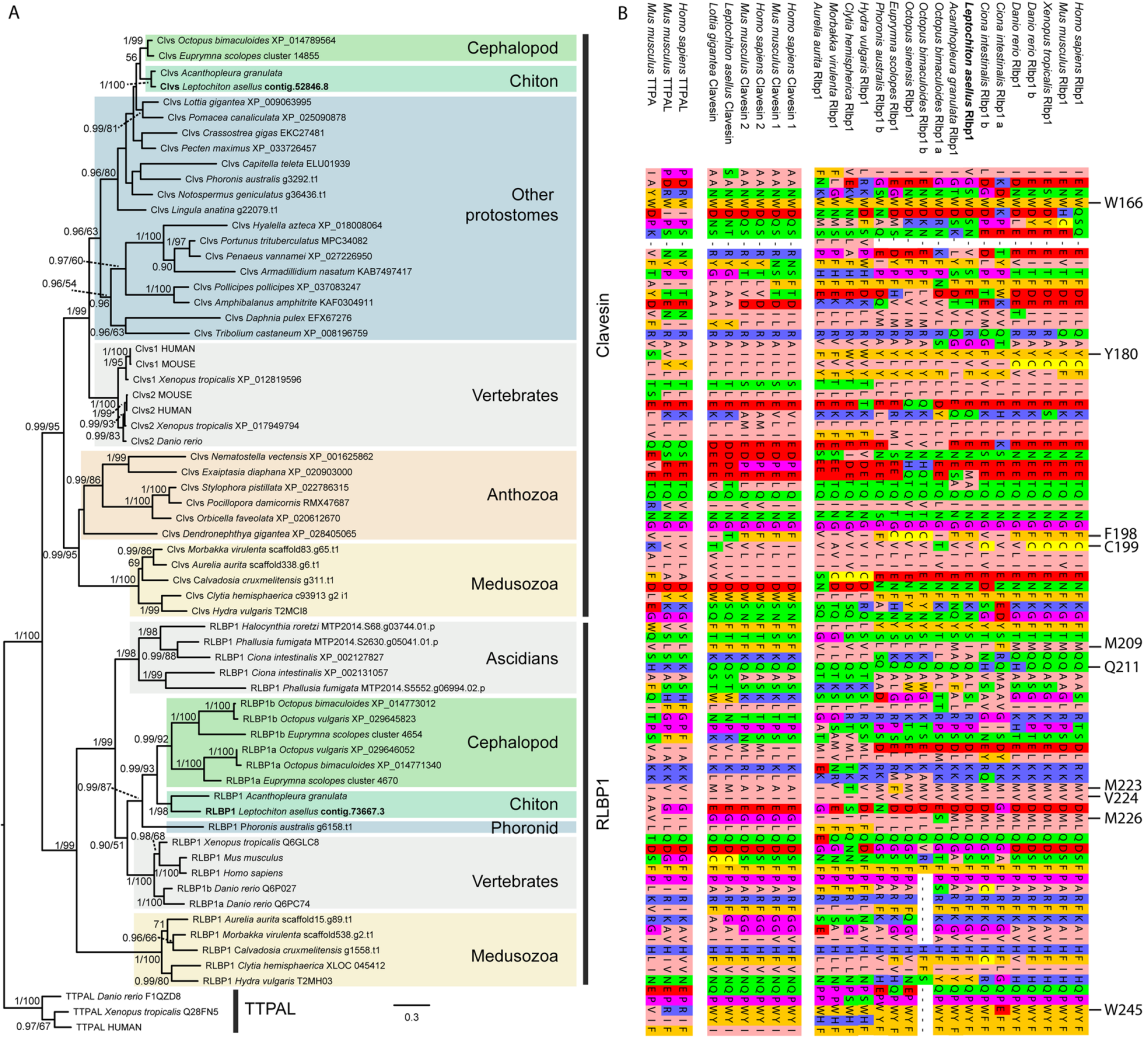
RLBP1 phylogeny and sequence alignment. (**A**) Maximum-likelihood tree (RAXML) of the RLBP1 and Clavesin families. Bayesian posterior probabilities (left value) higher than 0.90 and ML bootstrap (500 replicates, right value) higher than 50% are indicated below or above branches. (**B**) Conserved sites of known functional relevance in *RLBP1*. The amino acids W166, Y180, F198, C199, M209, Q211, M223, V224, M226, and W245 have been shown to be critically important for *RLBP1* to function. These amino acids are well-conserved among vertebrate *RLBP1s* but largely absent from *Clavesins* and *TTPAs*. *L.asellus RLBP1* exhibits 8 of 10 conserved residues

In order to further characterize the distribution of *RLBP1* in animals, we performed an extensive search of this gene in available genomes (see Additional file 6: Table S1) followed by phylogenetic analyses (Fig. 3A). We identified *RLBP1 *homologs in all checked medusozoan, cephalopod, chiton and phoronid genomes, but not in any anthozoans, non-chordate deuterostomes, bivalves, gastropods or those of other protostome groups. Conversely, we could identify *clavesin*, the closest relatives of *RLBP1*, from all major eumetaozan groups (including *L. asellus*) further strengthening the absence of *RLBP1* from most available invertebrate genomes (Fig. 3A). Phylogenetic analyses supported monophyly of RLBP1. Chordate RLBP1s were recovered as paraphyletic, with vertebrate RLBP1 grouping with protostome instead of ascidian sequences. This is likely the result of a reconstruction artefact as paraphyly of chordate RLBP1 is only weakly supported by Maximum Likelihood (ML bootstrap 51%) and Bayesian analyses (PP = 0.90) and is not significantly different from a tree showing monophyletic chordate RLBP1 (Approximately Unbiased (AU) test p = 0.437) [50]. Taken together these analyses strongly suggest a high number of losses of *RLBP1* in the invertebrates.

### Conserved sites of known functional relevance in Las-RLBP1

The function of RLBP1 and its active sites have been intensively studied in vertebrates. It binds 11-cis-retinol and facilitates its oxidation to 11-cis retinal by RDH5 in the dark cycle before the 11-cis retinal is transported back into the PRCs [4, 51, 52]. Ten amino acids have been identified to be critical for these tasks by site directed mutagenesis, i.e., W166, Y180, F198, C199, M209, Q211, M223, V224, M226, and W245 [52, 53]. Surprisingly, *Las-RLBP1* shows very high similarity with vertebrate RLBP1 by exhibiting eight of the ten critical amino acids (Fig. 3B), indicating potentially similar binding properties as described in vertebrates. In case of another chiton, *Acanthopleura granulata*, even nine out of the ten amino acids were conserved. The RLBP1 sequences from cephalopods are slightly less conserved, but particularly in the sequences of *Octopus bimaculoides* still six of the respective residues are present as well (Fig. 3B). Interestingly, the RLBP1 sequence found in the phoronid *Phoronis australis* has seven out of the ten conserved amino acids. In contrast, the RLBP1 sequences found in cnidarians exhibit only three out of the ten conserved functional sites of the vertebrate RLBP1 critical for retinoid binding [52, 53].

### Both retinal binding proteins are expressed in the PRCs of *Leptochiton asellus*

**Fig. 4:**
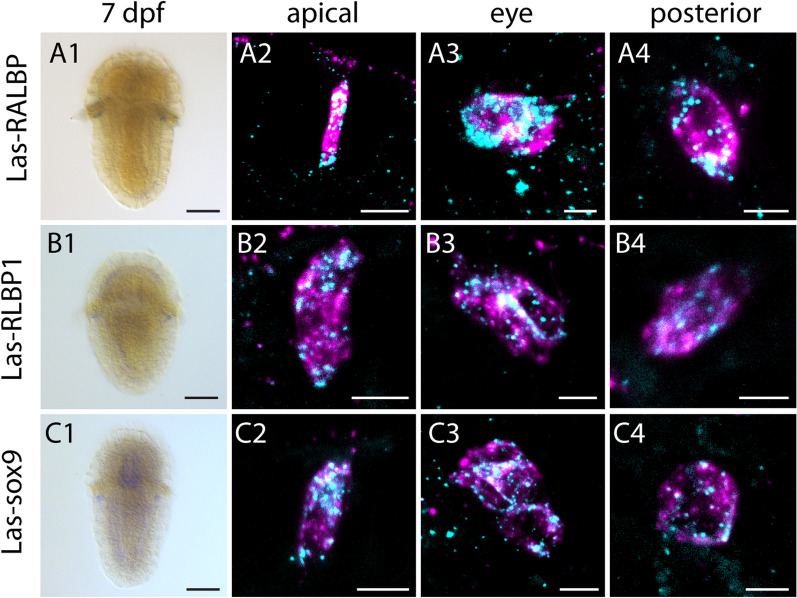
Expression of *L. asellus* retinoid binding proteins and *Las-sox9* in 7dpf larva. Column 1: single labeling of gene X. Column 2–4: double labeling of gene X (cyan) and *Las-r-opsin* (magenta) in the anterior, posttrochal eye and posterior region. *Las-RALBP* (**A1**–**A4**), *Las-RLBP1* (**B1**–**B4**) and *Las-sox9* (**C1**–**C4**) are co-expressed with *Las-r-opsin* in anterior, eye and posterior PRCs. Scale bars: 100 μm in column **1**; 5 μm in columns **2**–**4**

We performed in situ hybridization analysis in order to determine the expression patterns of the two retinal binding proteins (Fig. 4A, B). Both *Las-RALBP* and *Las-RLBP1* showed expression in the eye region, the apical area and the posterior body region of larvae similar to *Las-retinochrome*. Colocalization experiments with *Las-r-opsin* confirmed the co-expression with r-opsin and hence a co-expression with *Las-retinochrome* in all PRCs (Fig. 4A, B). This indicates a potential involvement in the retinal recycling process in the larval PRCs of both *Las-RALBP* and *Las-RLBP1*.

### Known regulators of vertebrate *RLBP1* are expressed in *L. asellus* photoreceptors

A recently published study on human and mouse RPE cells identified some transcription factors involved in the regulation of vertebrate RLBP1 [54]. It was proposed that sox9 and otx2 directly regulate RLBP1 but are regulated themselves by other factors including mitf and lhx2. *Las-otx2* and *Las-lhx2/9* have already been found to be expressed in all r-opsin + cells of *L. asellus*, whereas *Las-mitf* was expressed in the closely adjacent pigment cells of the larval eye [31]. Hence, we searched for *sox9* in our transcriptome data and identified a sequence, which was confirmed to be a homolog by subsequent phylogenetic analysis (Additional file 3: Fig. S3). We found *Las-sox9* to be co-expressed with *Las-r-opsin* in all PRCs indicating a potentially similar regulatory regulation of *RLBP1* between vertebrates and *L. asellus* (Fig. 4C).

### Retinal dehydrogenases in *L. asellus*

**Fig. 5:**
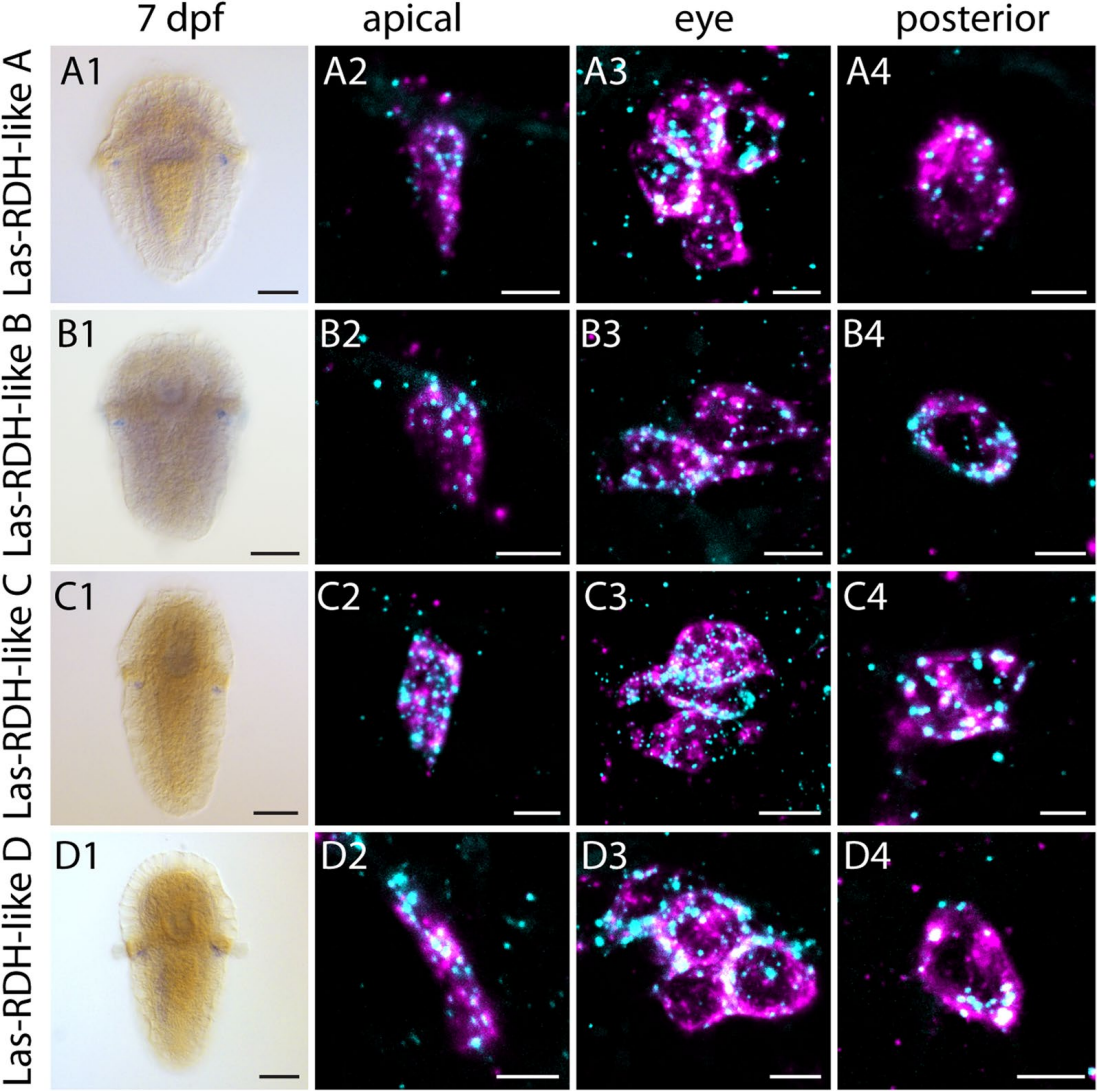
Expression of retinal dehydrogenases in *L. asellus* in 7dpf larva. Column 1: single labeling of gene X. Column 2–4: double labeling of gene X (cyan) and *Las-r-opsin* (magenta) in the anterior, post trochal eye and posterior region. *Las-RDH A* (**A1**–**A4**), *Las-RDH B* (**B1**–**B4**), *Las-RDH C* (**C1**–**C4**) and *Las-RDH D* (**D1**-**D4**) are co-expressed with *Las-r-opsin* in anterior, eye and posterior PRCs. Scale bars: 100 μm in column **1**; 5 μm in columns **2**–**4**

A group of proteins involved at several steps of the visual cycle of vertebrates and retinal recycling in *Drosophila* are retinal dehydrogenases (RDHs) [10]. We found five RDH candidate sequences A, B, C, D and E in our transcriptome. The sequences for RDH B-E appear to belong to the same clade, and thereby be closely related, whereas A is more distantly related (Additional file 4: Fig. S4). Four of these candidates, A, B, C and D, were found to be expressed in *L. asellus* PRCs (Fig. 5). However, due to the sheer number of different types of alcohol dehydrogenases, phylogenetic analysis has proven to be very difficult and delineation of orthology relationships is not possible based on our data. Though the observed RDHs may serve similar functions as described in vertebrates and *Drosophila*, the evolutionary significance remains speculative.

## Discussion

### The cephalopod rhodopsin-retinochrome system may be present in many protostomes

The cephalopod rhodopsin-retinochrome system has been analyzed in pioneering studies on protostome visual cycling, but it has not been described anywhere else and seemed to be a rather unique solution [16, 18–21]. As stated by Ozaki et al. [19] and Hara and Hara [16], key functions of the rhodopsin-retinochrome may be a parallel mechanism of retinal re-isomerization and a storage role for supplying the light detecting rhodopsin with 11-cis-retinal in dark conditions. Our results show that the fundamental elements of the cephalopod rhodopsin-retinochrome system, an r-opsin, retinochrome and the shuttle protein RALBP are all co-expressed in the PRCs of larval chitons. Sequence analyses, as well as the subcellular location of the specific Las-retinochrome and Las-r-opsin protein show further resemblance to the situation in cephalopod eye PRCs. The data strongly suggest that the rhodopsin-retinochrome system is not restricted to cephalopods but was present already in the last common ancestor of chitons and cephalopods, which likely is the last common ancestor of all living Mollusca [23–26]. In fact, our phylogenetic analysis of the retinochrome/RGR/peropsin clade confirms the findings of Ramirez et al. [22] that retinochromes can be found in several protostome animals. According to our phylogenetic analyses, the same holds true for the retinal binding protein RALBP suggesting that the whole machinery of the rhodopsin-retinochrome system may indeed be employed also in other protostomes. This also corroborates previous studies reporting the presence of the different components in gastropods based on antibody stainings [20, 21]. We could, however, not find RALBP orthologs in arthropods or vertebrates.

### Evolution and function of opsin photoisomerases

Corroborating earlier studies [39, 55, 56], our data support the idea of a monophyletic photoisomerase opsin group. Several subgroups are well-supported in our analysis, i.e., deuterostome RGRs and peropsins, protostome retinochromes and arthropod and mollusk peropsin-like opsins as it has also been found by Ramirez et al. [22]. The identification and inclusion of further retinochrome sequences may be an important factor for increased tree resolution and reduced considerably branch length of this clade, which has been a reason why retinochrome was excluded by other analyses [56]. The involvement of vertebrate RGRs and squid retinochrome in visual cycling is well-proven [5, 16, 57] and we suggest that other protostome retinochromes fulfill a similar function. Contribution to visual cycling has also been suggested for vertebrate peropsins [2, 58] as well as arthropod peropsin-like opsins [59, 60], though the detailed role in the process still has to be determined. Both are expressed in retinal pigment epithelium cells closely associated to PRCs and photoisomerase function is proposed. A function in visual cycling might thus be ancestral for the whole photoisomerase opsin family. In accordance with this idea, our sequence analyses indicate bistability as a common feature in all photoisomerase subgroups.

The only opsins where the key function to activate a G-protein mediated signaling cascade is assumed to be missing are RGRs and retinochromes [2, 39], while vertebrate peropsins and a mutated peropsin of a spider were suggested to have this capability [15, 40, 61]. In consistency, our sequence analyses show that most of the residues known to be crucial for G-protein binding and activation are conserved in deuterostome peropsins as it is common in other opsin types such as c-opsins and r-opsins. Most deviations occur in RGR and retinochrome sequences, but also arthropod and mollusk peropsin-like pigments show modifications. However, since the comparison between the sequences reveals that each group is modified in different positions, independent evolutionary modifying events appear likely. This is most evident for RGRs and retinochromes, each of which happen to be modified in multiple, but not identical positions. For example, in the NPXXY motif, N is modified in retinochromes and P in RGRs. In consequence, the ancestral opsin of the photoisomerase family most probably was capable of inducing phototransduction. This capability may still be present in peropsins, as a recent study suggests [61]. Our data, however, strongly suggest that this function has been lost independently in RGRs and retinochromes.

### Involvement of RLBP1 and RDHs in the visual cycle of mollusks

Prior to this study, RLBP1-like had been identified only in *Hydra* [7] but homology of this sequence was put into question due to lack of critical binding sites and absence of RLBP1 homologs in other non-chordate animals [7, 9]. Through genome mining and phylogenetic analyses, we could identify RLBP1 homologs in all medusozoan classes as well as in cephalopods, chitons and phoronids with high degree of conservation of the functional binding sites. We could not find RLBP1 in any other animal groups, suggesting multiple gene losses of this gene during evolution. *Las-RLBP1* was found to be exclusively expressed in PRCs, strongly suggesting participation of this gene in the retinoid metabolism and visual cycling. Functional studies will be required to assess whether *Las-RLBP1* contributes to the rhodopsin-retinochrome system and/or participates in a so far undescribed light-independent dark cycle. The expression of the transcription factors *Las-sox9*, *Las-otx2* and *Las-lhx2/9* in the *L. asellus* PRCs suggests similarities to the regulation of *RLBP1* expression in vertebrates [54]. These data strongly suggest that *RLBP1* and its participation in visual cycling may predate the origin of vertebrates.

We could identify four RDH genes expressed in the *L. asellus* PRCs. This suggests their involvement in the visual cycle, confirming previous results obtained in other protostome species [10, 11]. Involvement of RDHs in the visual cycle of cephalopods and insects had indeed been suggested previously [4, 62]. In Drosophila, RDHs were shown to prevent the toxic effects of retinal [4, 63, 64]. Functional studies will be necessary to determine the exact role of RDH in the mollusk visual systems.

### Involvement of Xenopsin in *Leptochiton asellus* retinal re-isomerization

The finding of a second signaling opsin, xenopsin (originally annotated as ciliary opsin by Yoshida et al. [65]), expressed in *L. asellus* PRCs [30] and in cephalopod eyes [65], suggest further complexity of the visual cycling process of mollusks. The counterions in Las-xenopsin are in positions suggesting monostability of this opsin, implicating that one opsin in the PRCs of *L. asellus* would completely rely on external retinal re-isomerization. This likewise could be covered by photoisomerase activity of *Las-retinochrome* and the shuttle *Las-RALBP*. Alternatively, *Las-xenopsin* might depend on an undescribed dark cycle, potentially involving *Las-RLBP1*. As a consequence, further studies on mollusk visual cycling will be important to deepen the understanding of invertebrate visual cycling.

## Conclusion

This is the first analysis providing unambiguous evidence for the presence of a retinochrome in r-opsin expressing PRCs outside cephalopods. We further found the retinal shuttle *RALBP* to be co-expressed with *r-opsin* in chiton. Protein distribution of these two opsins within the PRCs resembles the situation in cephalopods. Accordingly, we suggest that employment of retinochrome and RALBP in retinal re-isomerization is not limited to cephalopods but is a general feature of molluscan r-opsin + PRCs. Taxonomic distribution of RALBP and retinochromes suggest the presence of the rhodopsin-retinochrome system in other protostome animals. Additionally, we suggest that molluscan visual cycling is more complex than postulated so far, due to the additional finding of *Las-Rlbp1*, a homolog of a vertebrate retinal binding protein and the co-expression of *Las-xenopsin* all in the same PRCs. Existence of a dark cycle supplying a potentially monostable xenopsin is conceivable. Our detailed sequential analysis of *RLBP1* suggests functional similarities to the vertebrate enzyme, indicating this particular element of the vertebrate visual cycle might also play a role in the visual cycle of protostomes.

## Methods

### *Leptochiton asellus* culture

Adult animals of *L. asellus* were collected during September-December 2011–2014 near the coast of Bergen in Norway. The animals were kept in groups of males and females in tanks with filtered natural seawater at 8 °C without direct light exposure and fertilized eggs were collected each morning. Lecitotrophic larvae were raised in small bowls containing filtered seawater at 18 °C on a light/dark cycle of 12 h /12 h, without additional feeding.

### Gene cloning and RNA probe generation

The RNA-seq assembly of larval *L. asellus* described in Vöcking et al. [30, 31] was used for bidirectional blast (tblastn and blastx) to identify contiq sequences for genes of interest in the transcriptome data set [66, 67]. Subsequently, whole transcripts or fragments were amplified by means of PCR with specifically designed primers (Additional file 6: Table S2) and cDNA prepared with Super Script III (Invitrogen), ligated into pgemT-easy vector (Promega) and cloned into Top10 chemically competent *E. coli* (Invitrogen). Sanger sequencing was used to verify the cloned sequences before DIG- and FITC-labeled sense and antisense probes were generated with T7- and SP6-RNA Polymerases (Roche) or with Megascript Kit (Ambion).

### Sequence analysis and phylogenetic tree reconstruction

Public databases (Genbank, JGI, Uniprot) and the *Leptochiton* transcriptome were screened for homologs by text search, blast (blastp and tblastn) and HMMER (hmmscan and hmmseaarch) with respective query sequences or domain profiles for subsequent gene tree generation [66–68]. Sequences were aligned with MAFFT 7.221 (EINSI option) and manually curated to remove gaps and regions of low alignment quality [69]. Best fitting substitution models were selected with ProtTest 3.4 according to Akaike information, Bayesian information and Decision theory criteria or with the RAXML based ProteinModelSelection script [70, 71]. Maximum likelihood analyses were run with RAXML 8.2.8 on the CIPRES Science Gateway [72] with gamma model of rate heterogeneity and rapid bootstrapping with automatic stop by majority rule criterion autoMRE. Bayesian analyses were run with Phylobayes 4.1 or Phylobayes-MPI 1.6 using the same substitution models chosen for ML analyses and gamma distribution for rate heterogeneity [73–75]. At least three chains were run for 50.000 cycles and tested for convergence and mixing behavior with bcomp (maxdiff < 0.1) and tracecomp (minimum effective size > 3000) command discarding the 20% first cycles as burn-in and used for generating majority-rule consensus trees. In case of the opsin phylogeny, a data-set specific substitution matrix (HM-GTR) was prepared by optimizing GTR model parameters based on a large opsin sequence sampling [56]. HM-GTR + Γ was used for both RAXML and Phylobayes analyses. For sequence analysis and comparison of motifs, sequences were studied with the Jalview Version 2.7 [76].

To perform the approximately unbiased test [50], the likelihood of the best ML topology obtained with RaxML (model LG + Γ (8)) was compared with the best topology under the constraint of monophyly of RLBP1 from all chordates using CONSEL [77]. Branch lengths were estimated using Tree Puzzle 5.3 under LG + Γ(8) model [78].

### Immunohistochemistry

Custom-made polyclonal antibodies against the peptides AMATTDQAVIDDSHI and IRASSATDTDISDKVNP from the N-terminal extracellular end and the cytosolic loop V of *Las-retinochrome* were raised in rats and affinity purified by 21st Century Biochemicals (Marlboro, USA). A blast search (tblastn) was performed for both peptide sequences against the *Leptochiton* transcriptome, to assure antigen specificity. This resulted in the single existent retinochrome as the only hit. Only the antibody affinity raised against the AMATTDQAVIDDSHI gave clear signals and was used for the experiments. Dot blot experiments and preadsorption negative controls were performed to ensure antibody specificity (Additional file 5: Fig. S5). Las-r-opsin antibody was described by Vöcking et al. [31]. Stainings were performed as described previously [31]. Briefly, after fixation in 4% PFA in buffer PBS (0.05 M PB/0.3 M NaCl/0.1% Tween; pH 7.4) samples were washed and stored in PBS. For the staining procedure samples were transferred to THT (0.1 M Tris, 0.1% Tween 20). Primary antibodies were applied for 48–72 h on 4 °C, diluted 1:100 in THT with 5% sheep serum. After subsequent washing the secondary antibodies were applied for 24 h on 4 °C. Samples were stored in embedding medium (90% glycerol/1 × PBS/ 0.25% DABCO) until usage.

### In situ hybridization

Experiments were performed as described previously [30, 31]. Briefly, animals were fixed in 2.5% PFA in phosphate buffer and with Tween20 (PTW; pH 7.4) and subsequently washed and stored in Methanol. After stepwise rehydration in PTW, samples were shortly digested with Proteinase K, washed and prehybridized in hybridization buffer. Hybridization lasted 72 h and stainings were done with a combination of Fast Blue (Sigma-Aldrich) and Fast Red (Roche). To evaluate the significance of the stainings both, sense and antisense probes were used and no staining was obtained from sense probes. In addition, in situ hybridization and antibody stainings were combined by performing the in situ protocol and processing the samples afterwards with the aforementioned immunohistochemistry procedure.

### Light microscopy

Light microscopic images were taken using a Nikon Eclipse E800 and a Nikon AZ100M microscope and adjusted with Photoshop CS5. Confocal images were taken with a Leica SP5 confocal microscope and the image stacks processed with ImageJ and Photoshop CS5.

## Supplementary Information

**Additional file 1: Fig. S1.** Phylogenetic analysis of opsin photoisomerase family. Maximum-likelihood tree inferred with RAXML using an opsin specific substitution matrix as model (HM-GTR + Γ). Bootstrap values and Bayesian posterior probabilities of Bayesian analysis (Phylobayes, HM-GTR + Γ, 3 chains, 50.000 cycles, 20% burn-in) are given as support values.

**Additional file 2: Fig. S2.** Phylogeny of retinoid binding proteins. Maximum-likelihood (RAXML, LG + Γ). Bootstrap values and Bayesian posterior probabilities of Bayesian analysis (Phylobayes, LG + Γ, 3 chains, 55.000 cycles, 20% burn-in) are given as support values.

**Additional file 3: Fig. S3.** Sox9 evolution Maximum-likelihood (RAXML, LG + Γ + F model, rapid bootstrapping (250 replicates)).

**Additional file 4: Fig. S4.** Phylogeny of Retinal dehydrogenases. Maximum-likelihood (RAXML, LG + Γ model, rapid bootstrapping (250 replicates)).

**Additional file 5: Fig. S5.** Retinochrome antibody preadsorption test. (**A1**-**3**) The negative control of the specifically designed *retinochrome *antibody shows no specific signal in the eye region after the specimen and antibody were preadsorped with the antigenic peptide AMATTDQAVIDDSHI (0.25 mg/ml). **(B1-3)** The positive control of the antibody shows a clear signal in the eye region (Scalebars: 15 μm in **A**; 5 μm in **B**).

**Additional file 6: Table S1.** Search for *RLBP1* in metazoan genomes and transcriptomes. **Table S2.** Primer sequences used to generate RNA probes for in situ hybridization.

## Data Availability

The sequence data are available at the European Nucleotide Archive (Accession numbers: OD960290-OD960298; https://www.ebi.ac.uk/ena/browser/view/OD960290-OD960298).

## Abbreviations

PRC: Photoreceptor cells
RDH: Retinal dehydrogenases
RPE: Retinal pigment epithelium
