# Supplementary figures and images for: The rhodopsin-retinochrome system for retinal re-isomerization predates the origin of cephalopod eyes
Source: BMC Ecol Evol. 2021 Nov 29;21:215. doi: 10.1186/s12862-021-01939-x (PMC8628405; doi:10.1186/s12862-021-01939-x)

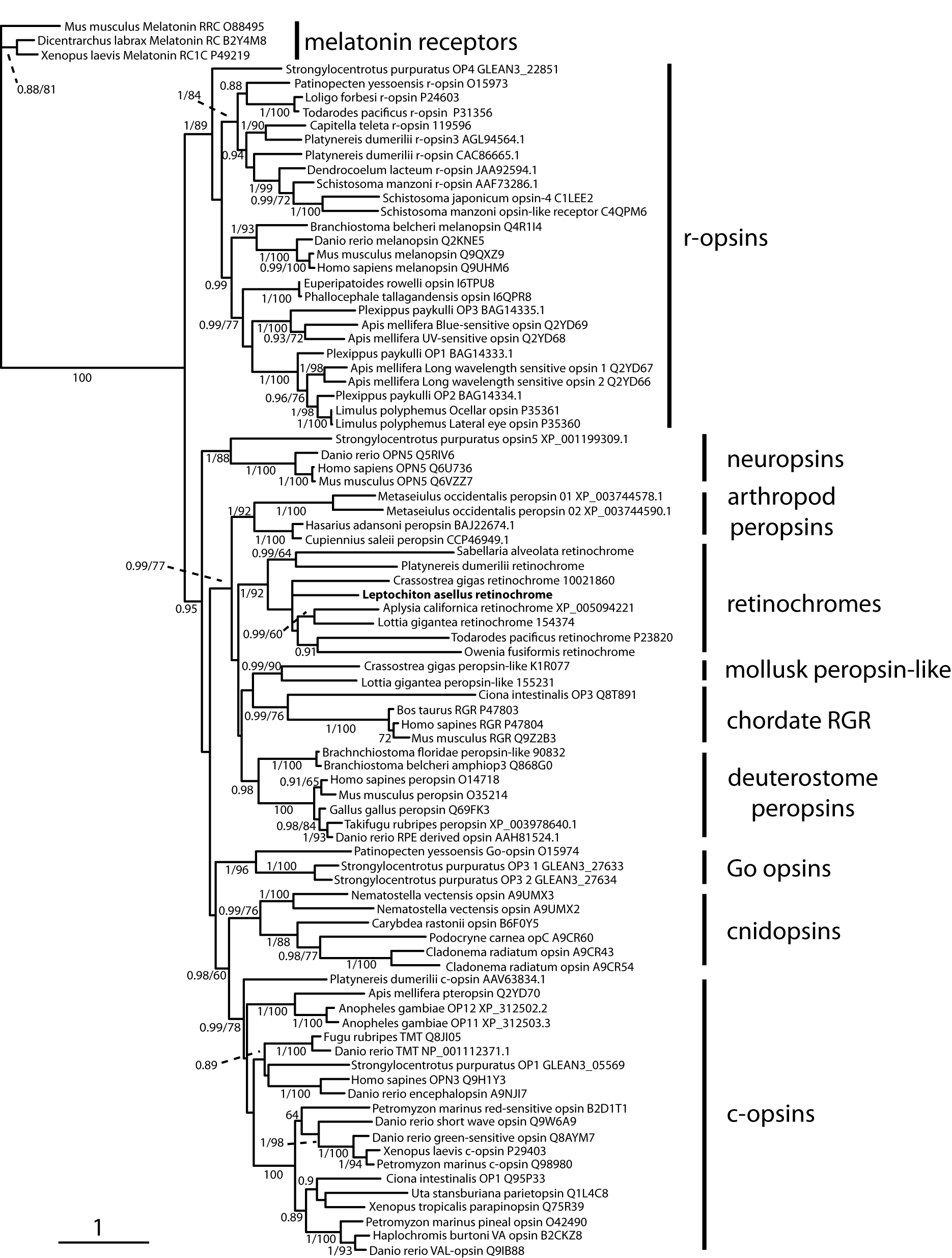

Supplement: Supplementary file 1 — Additional file 1: Fig. S1. Phylogenetic analysis of opsin photoisomerase family. Maximum-likelihood tree inferred with RAXML using an opsin specific substitution matrix as model (HM-GTR + Γ). Bootstrap values and Bayesian posterior probabilities of Bayesian analysis (Phylobayes, HM-GTR + Γ, 3 chains, 50.000 cycles, 20% burn-in) are given as support values. [file 12862_2021_1939_MOESM1_ESM.tif]

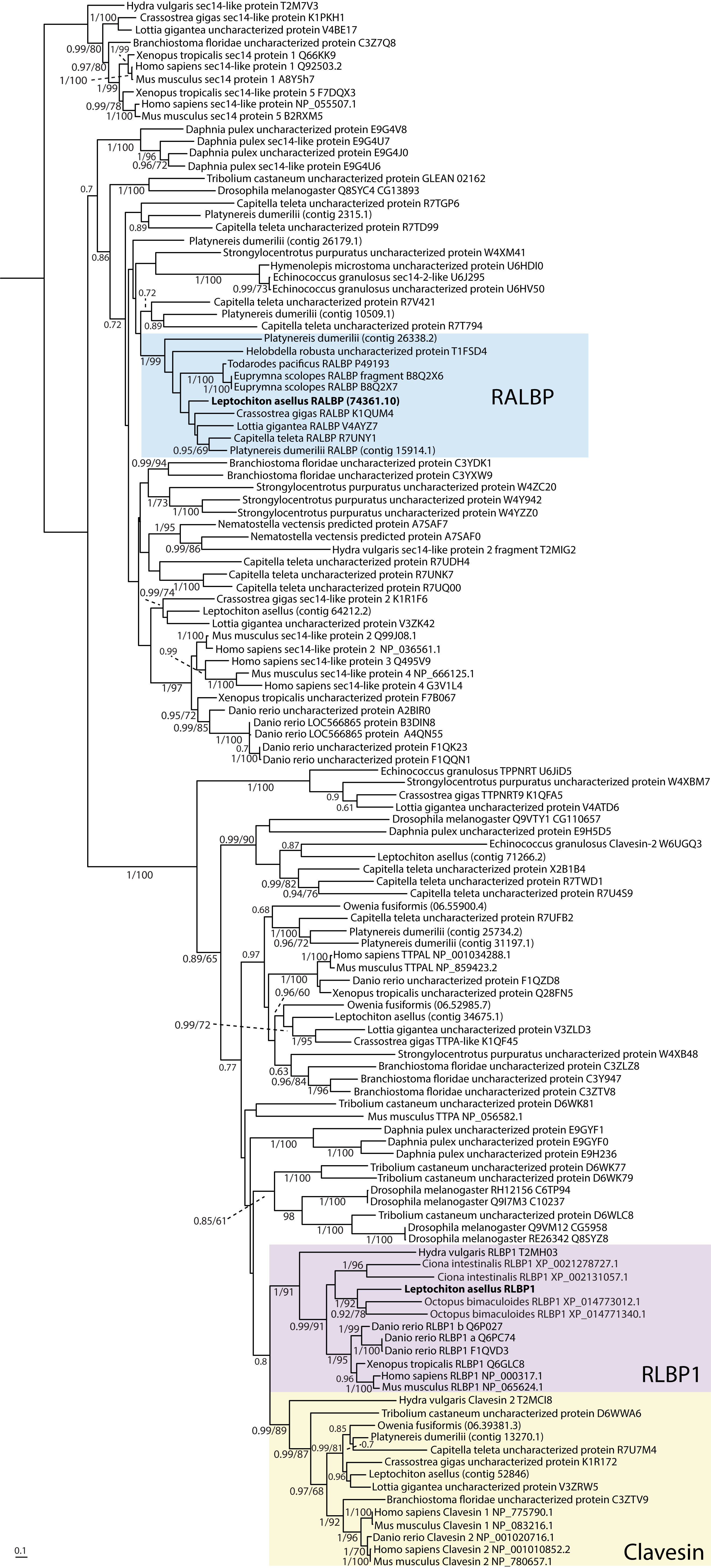

Supplement: Supplementary file 2 — Additional file 2: Fig. S2. Phylogeny of retinoid binding proteins. Maximum-likelihood (RAXML, LG + Γ). Bootstrap values and Bayesian posterior probabilities of Bayesian analysis (Phylobayes, LG + Γ, 3 chains, 55.000 cycles, 20% burn-in) are given as support values. [file 12862_2021_1939_MOESM2_ESM.tif]

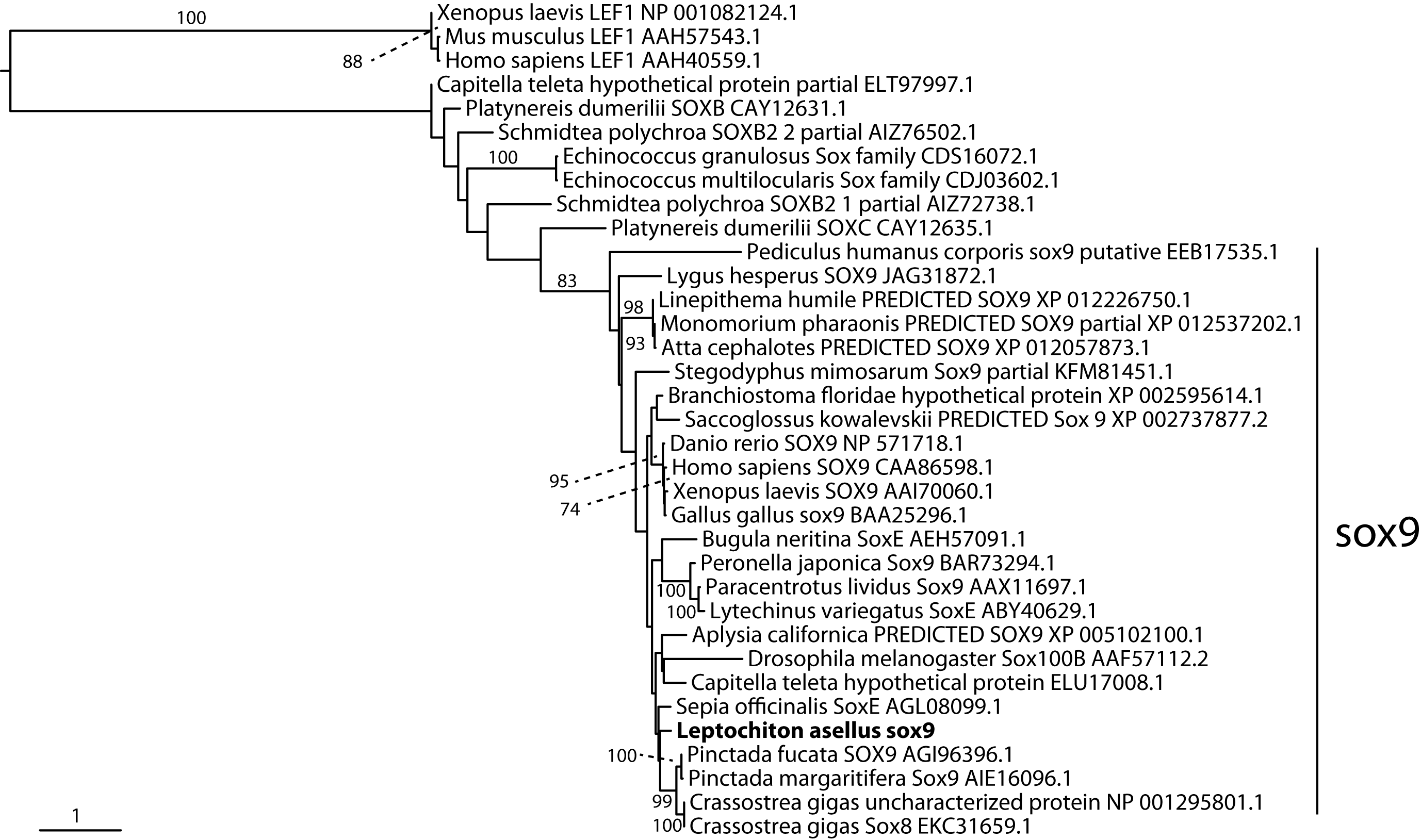

Supplement: Supplementary file 3 — Additional file 3: Fig. S3. Sox9 evolution Maximum-likelihood (RAXML, LG + Γ + F model, rapid bootstrapping (250 replicates)). [file 12862_2021_1939_MOESM3_ESM.tif]

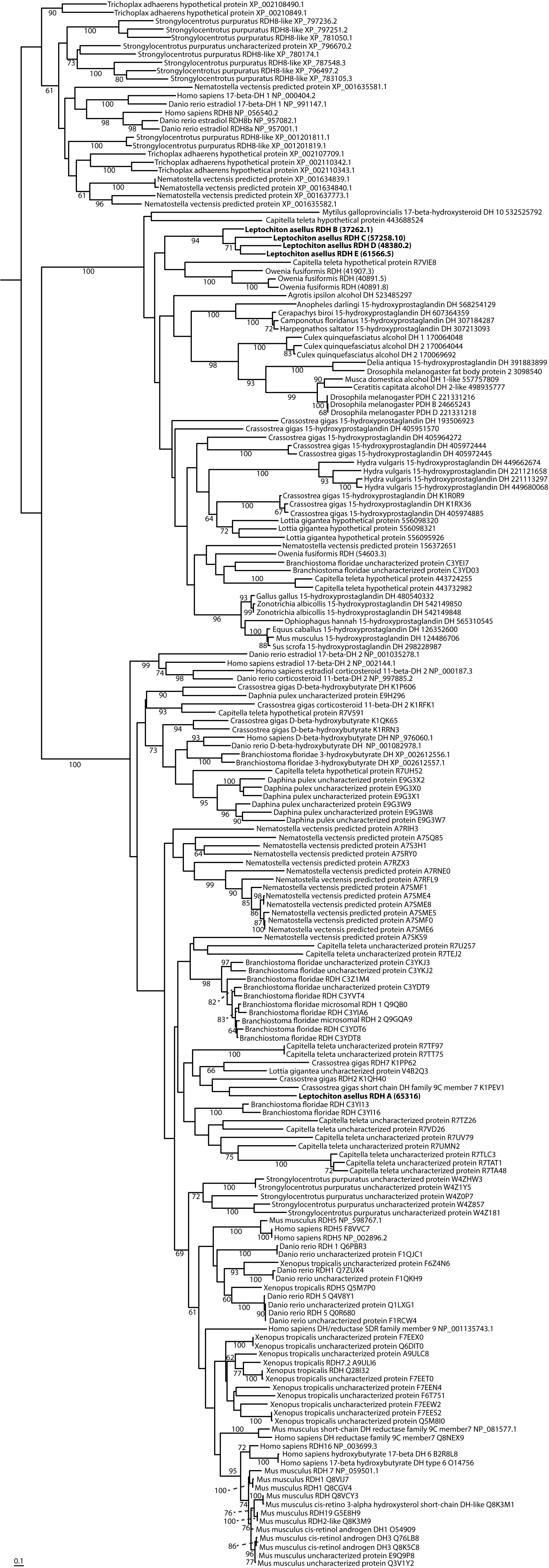

Supplement: Supplementary file 4 — Additional file 4: Fig. S4. Phylogeny of Retinal dehydrogenases. Maximum-likelihood (RAXML, LG + Γ model, rapid bootstrapping (250 replicates)). [file 12862_2021_1939_MOESM4_ESM.tif]

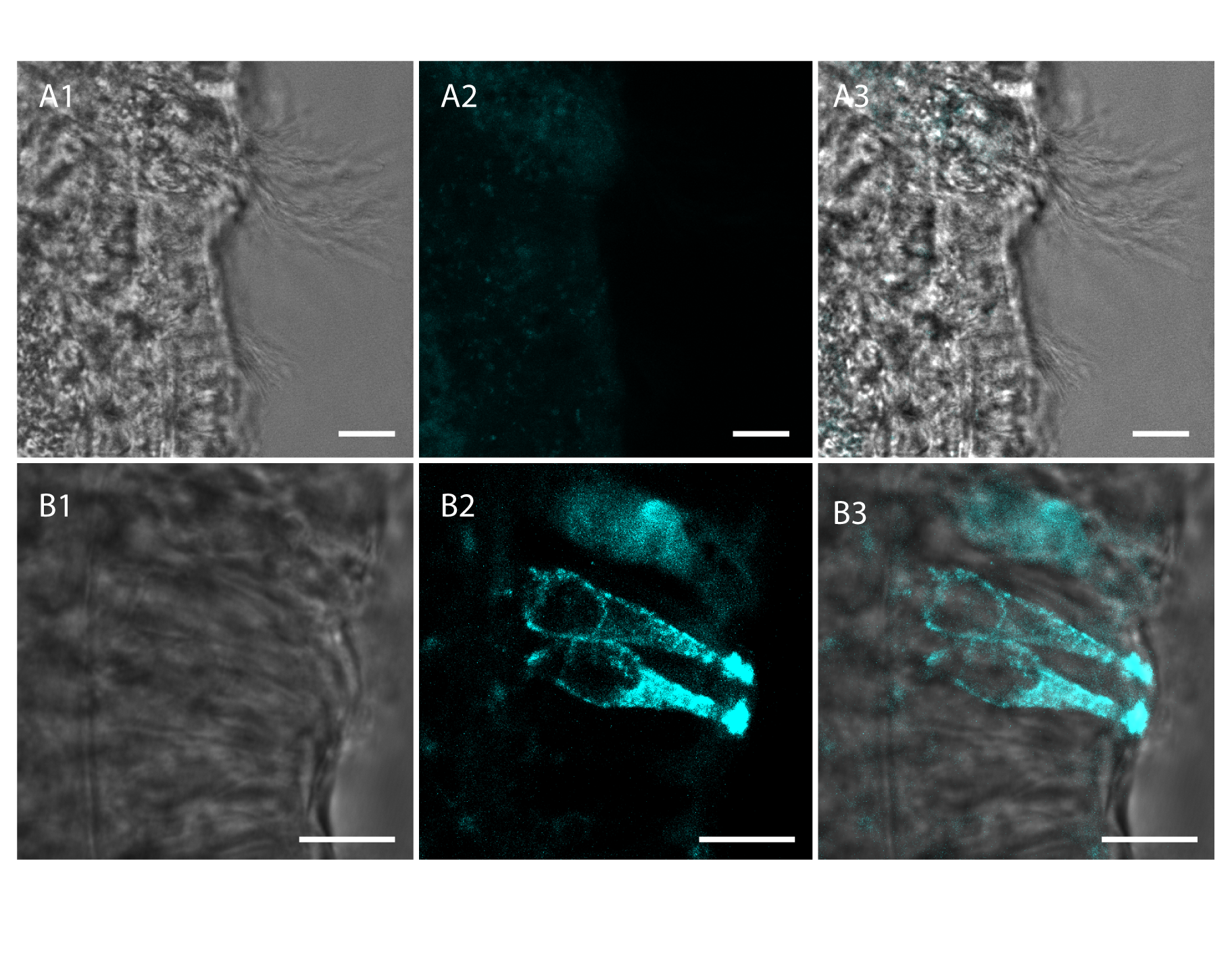

Supplement: Supplementary file 5 — Additional file 5: Fig. S5. Retinochrome antibody preadsorption test. (A1-3) The negative control of the specifically designed retinochrome antibody shows no specific signal in the eye region after the specimen and antibody were preadsorped with the antigenic peptide AMATTDQAVIDDSHI (0.25 mg/ml). (B1-3) The positive control of the antibody shows a clear signal in the eye region (Scalebars: 15 μm in A; 5 μm in B). [file 12862_2021_1939_MOESM5_ESM.tif]
