# Supplementary material for: The rhodopsin-retinochrome system for retinal re-isomerization predates the origin of cephalopod eyes
Source: BMC Ecol Evol. 2021 Nov 29;21:215. doi: 10.1186/s12862-021-01939-x (PMC8628405; doi:10.1186/s12862-021-01939-x)
Supplement: Supplementary file 6 — Additional file 6: Table S1. Search for RLBP1 in metazoan genomes and transcriptomes. Table S2. Primer sequences used to generate RNA probes for in situ hybridization. [file 12862_2021_1939_MOESM6_ESM.docx]

**Supplementary Material**

**The rhodopsin-retinochrome system for retinal re-isomerization predates the origin of cephalopod eyes**

Oliver Vöcking, Lucas Leclère and Harald Hausen

**Table S1. Search for *RLBP1* in metazoan genomes and transcriptomes.**

| **Clade** | **Species** | **RLBP1** | **Blasted data** | **Source** |
| --- | --- | --- | --- | --- |
| Ctenophora | *Pleurobrachia pileus* | Not found | Genome | neurobase.rc.ufl.edu/pleurobrachia |
| Ctenophora | *Mnemiopsis leidyi* | Not found | Genome | Ensembl Metazoa |
| Demospongiae | *Amphimedon queenslandica* | Not found | Genome | Ensembl Metazoa |
| Demospongiae | *Ephydatia muelleri* | Not found | Genome | spaces.facsci.ualberta.ca/ephybase |
| Demospongiae | *Stylissa carterri* | Not found | Genome | compagen.org |
| Homoscleromorpha | *Oscarella carmela* | Not found | Genome | compagen.org |
| Calcarea | *Sycon ciliatum* | Not found | Genome | compagen.org |
| Placozoa | *Trichoplax adhaerens* | Not found | Genome | Ensembl Metazoa |
| Myxozoa | *Thelohanellus kitauei* | Not found | Genome | Ensembl Metazoa |
| Anthozoa | *Nematostella vectensis* | Not found | Genome | Ensembl Metazoa |
| Anthozoa | *Exaiptasia pallida* | Not found | Genome | NCBI nr / NCBI genome |
| Anthozoa | *Acropora digitifera* | Not found | Genome | NCBI nr / NCBI genome |
| Anthozoa | *Orbicella faveolata* | Not found | Genome | NCBI nr / NCBI genome |
| Anthozoa | *Stylophora pistillata* | Not found | Genome | NCBI nr / NCBI genome |
| Anthozoa | *Dendronephthya gigantea* | Not found | Genome | NCBI nr / NCBI genome |
| Hydrozoa | *Hydra vulgaris* | **Present** | Genome | research.nhgri.nih.gov/hydra |
| Hydrozoa | *Clytia hemisphaerica* | **Present** | Genome | marimba.obs-vlfr.fr |
| Scyphozoa | *Aurelia aurita* | **Present** | Genome | marinegenomics.oist.jp |
| Cubozoa | *Morbakka virulenta* | **Present** | Genome | marinegenomics.oist.jp |
| Staurozoa | *Calvadosia cruxmelitensis* | **Present** | Genome | NCBI nr / NCBI genome |
| Acoela | *Praesagittifera naikaiensis* | Not found | Genome | marinegenomics.oist.jp |
| Echinodermata | *Acanthaster planci* | Not found | Genome | NCBI nr / NCBI genome |
| Echinodermata | *Strongylocentrotus purpuratus* | Not found | Genome | NCBI nr / NCBI genome |
| Echinodermata | *Apostichopus japonicus* | Not found | Genome | NCBI nr / NCBI genome |
| Hemichordata | *Saccoglossus kowalevskii* | Not found | Genome | NCBI nr / NCBI genome |
| Hemichordata | *Ptychodera flava* | Not found | Genome | marinegenomics.oist.jp |
| Cephalochordata | *Branchiostoma floridae* | Not found | Genome | JGI MycoCosm |
| Cephalochordata | *Branchiostoma lanceolatum* | Not found | Genome | marimba.obs-vlfr.fr |
| Urochordata | *Ciona intestinalis* | **Present** | Genome | NCBI nr / aniseed.cnrs.fr |
| Urochordata | *Ciona savignyi* | **Present** | Genome | aniseed.cnrs.fr |
| Urochordata | *Phallusia mammillata* | **Present** | Genome | aniseed.cnrs.fr |
| Urochordata | *Phallusia fumigata* | **Present** | Genome | aniseed.cnrs.fr |
| Urochordata | *Corella inflata* | **Present** | Genome | aniseed.cnrs.fr |
| Urochordata | *Halocynthia roretzi* | **Present** | Genome | aniseed.cnrs.fr |
| Urochordata | *Molgula oculata* | **Present** | Genome | aniseed.cnrs.fr |
| Petromyzontiformes | *Petromyzon marinus* | **Present** | Genome | Ensembl |
| Gnathostomes | *taxid: 7776* | **Present** | nr/nt | NCBI - 22/03/2021 |
| Priapulida | *Priapulus caudatus* | Not found | Genome | NCBI nr / NCBI genome |
| Nematoda | *taxid: 6231* | Not found | nr/nt/TSA | NCBI - 22/03/2021 |
| Arthropoda | *taxid: 6656* | Not found | nr/nt/TSA | NCBI - 22/03/2021 |
| Onychophora | *taxid: 27563* | Not found | nr/nt/TSA | NCBI - 22/03/2021 |
| Tardigrade | *Hypsibius dujardini* | Not found | Genome | NCBI Genome |
| Platyhelminthes | *Schmidtea mediterranea* | Not found | Genome | smedgd.stowers.org |
| Platyhelminthes | *Macrostomum lignano* | Not found | Genome | parasite.wormbase.org |
| Brachiopoda | *Lingula anatina* | Not found | Genome | marinegenomics.oist.jp |
| Bryozoa | *Bugula neritina* | Not found | Genome | NCBI genome |
| Phoronida | *Phoronis australis* | **Present** | Genome | marinegenomics.oist.jp |
| Nemertea | *Notospermus geniculatus* | Not found | Genome | marinegenomics.oist.jp |
| Annelida | *Platynereis dumerilii* | Not found | Transcriptome | NCBI nr |
| Annelida | *Capitella teleta* | Not found | Genome | Ensembl Metazoa |
| Annelida | *Helobdella robusta* | Not found | Genome | Ensembl Metazoa |
| Rotifera | *Adineta vaga* | Not found | Genome | Ensembl Metazoa |
| Polyplacophora | *Leptochiton asellus* | **Present** | Transcriptome | This study |
| Polyplacophora | *Acanthopleura granulata* | **Present** | Genome | NCBI genome |
| Gastropoda | *Biomphalaria glabrata* | Not found | Genome | Ensembl Metazoa |
| Gastropoda | *Elysia chlorotica* | Not found | Genome | NCBI genome |
| Gastropoda | *Pomacea canaliculata* | Not found | Genome | NCBI genome |
| Bivalvia | *Bathymodiolus platifrons* | Not found | Genome | NCBI genome |
| Bivalvia | *Crassostrea gigas* | Not found | Genome | Ensembl Metazoa |
| Bivalvia | *Dreissena rostriformis* | Not found | Genome | NCBI genome |
| Bivalvia | *Lottia gigantea* | Not found | Genome | Ensembl Metazoa |
| Bivalvia | *Limnoperna fortunei* | Not found | Genome | NCBI genome |
| Bivalvia | *Modiolus philippinarum* | Not found | Genome | NCBI genome |
| Bivalvia | *Mytilus galloprovincialis* | Not found | Genome | NCBI genome |
| Bivalvia | *Mizuhopecten yessoensis* | Not found | Genome | NCBI genome |
| Bivalvia | *Pecten maximus* | Not found | Genome | NCBI genome |
| Bivalvia | *Pinctada fucata* | Not found | Genome | marinegenomics.oist.jp |
| Bivalvia | *Ruditapes philippinarum* | Not found | Genome | NCBI genome |
| Bivalvia | *Saccostrea glomerata* | Not found | Genome | NCBI genome |
| Bivalvia | *Venustaconcha ellipsiformis* | Not found | Genome | NCBI genome |
| Cephalopoda | *Euprymna scolopes* | **Present** | Genome | NCBI genome |
| Cephalopoda | *Hapalochlaena maculosa* | **Present** | Genome | NCBI genome |
| Cephalopoda | *Callistoctopus minor* | **Present** | Genome | cephalopodresearch.org/ceph_gdatab |
| Cephalopoda | *Octopus bimaculoides* | **Present** | Genome | cephalopodresearch.org/ceph_gdatab |
| Cephalopoda | *Octopus vulgaris* | **Present** | Genome | NCBI genome |
| Cephalopoda | *Sepia pharaonis* | **Present** | nr/nt | NCBI nr |

**Table S2** Primer sequences used to generate RNA probes for *in situ* hybridization

| **Primer** | **Species** | **Sequence** |
| --- | --- | --- |
| Las-ropsin Forward | *Leptochiton asellus* | CATCGACCGTTTTCTC |
| Las-ropsin Reverse | *Leptochiton asellus* | CATCTCCTCTAGTCGT |
| Las-retinochrome Forward | *Leptochiton asellus* | TGTGGGTGTAATGTATATGG |
| Las-retinochrome Reverse | *Leptochiton asellus* | ATGCTAGGAAGTTGGGT |
| Las-RALBP Forward | *Leptochiton asellus* | TTGTCCAAGTATCAGC |
| Las-RALBP Reverse | *Leptochiton asellus* | TATGTTCCATCCTCTGT |
| Las-RLBP1 Forward | *Leptochiton asellus* | GTCAAATATCGAGACAG |
| Las-RLBP1 Reverse | *Leptochiton asellus* | CTACATTCCACACAGT |
| Las-sox9 Forward | *Leptochiton asellus* | ATGAGCGATAGTGAAG |
| Las-sox9 Reverse | *Leptochiton asellus* | TAGGAGGTGTGAGTGT |
| Las-RDH A Forward | *Leptochiton asellus* | TTCGCCATAAACTCTC |
| Las-RDH A Reverse | *Leptochiton asellus* | TATGTCTACGCACCAA |
| Las-RDH B Forward | *Leptochiton asellus* | TCTTACATGGCGAGGA |
| Las-RDH B Reverse | *Leptochiton asellus* | TGTAACAAGAGCGAAC |
| Las-RDH C Forward | *Leptochiton asellus* | CAGCTTGAACATACAC |
| Las-RDH C Reverse | *Leptochiton asellus* | TTGCACATCTCACTAC |
| Las-RDH D Forward | *Leptochiton asellus* | AGTTAAAGGTGCGAAGG |
| Las-RDH D Reverse | *Leptochiton asellus* | CTGTGTCTAGGTGGATG |
